# Characteristics of sustained attention impairment in individuals under simulated positive acceleration stress: separation effect of early and late stage cognitive processing

**DOI:** 10.3389/fnbeh.2026.1733246

**Published:** 2026-08-13

**Authors:** Yang Liao, Yuyang Zhu, Rong Lin, Miao Jin, Yan Zhang, Yishuang Zhang, Liu Yang

**Affiliations:** Air Force Medical Center, Air Force Medical University, Beijing, China

**Keywords:** EEG, ERP, positive acceleration stress, sustained attention ability, time frequency analysis

## Abstract

**Objective:**

This study aimed to investigate the mechanisms underlying impaired sustained attention under positive acceleration stress.

**Methods:**

We used a lower body negative pressure model to simulate positive acceleration stress and employed electroencephalography (EEG) to observe changes in brain function.

**Results:**

EEG results indicated that sustained attention exhibited a dissociation effect between early and late stages of processing following positive acceleration stress. The latency of the P2 component induced by non-target stimuli at the Cz electrode was significantly reduced under lower body negative pressure conditions, suggesting enhanced early perceptual processing of visual stimuli under acceleration stress. The amplitude of the P300 component induced by non-target stimuli at the Pz and Cz electrodes was significantly lower than that at baseline, suggesting impaired late-stage response selection processes. These findings indicate that individuals exhibit adaptive capacity under cerebral ischemia induced by acceleration stress, whereby enhanced early perceptual processing may partially compensate for impairments in the late-stage response selection phase.

**Conclusion:**

The dissociation between the early and late stages of visual attention processing reflects the body’s adaptive adjustment ability in response to acceleration stress.

## 1 Introduction

Special occupational groups such as pilots and astronauts experience acceleration stress during specific stages of their missions (Clément et al., 2001; Fong and Fan, 1997; Pollock et al., 2024). Positive acceleration stress (+Gz) can cause blood to shift from the head toward the lower extremities, leading to acute cerebral ischemia and impaired cognitive and physical performance (Biernacki et al., 2013; Croft et al., 2021; Jia et al., 2009; McKinley et al., 2013; McKinlly and Gallimore, 2013; Rickards and Newman, 2005; Sandor et al., 2004; Truszczynski et al., 2014; Truszczynski et al., 2013). In severe cases, it can even cause loss of consciousness and compromise operational safety (Metzler, 2020). Understanding the physiological and psychological changes that occur under +Gz by cognitive science methods can help clarify the mechanisms underlying performance impairment and provide quantitative indicators for safety risk assessment and personnel selection in special occupational settings (Bacevic et al., 2024; Kim et al., 2017; Ryoo et al., 2004).

In theory, real-time observation of physiological and psychological changes experienced by pilots under +Gz during flight using onboard or wearable devices would provide the most reliable data (Croft et al., 2021; Kim et al., 2017). However, because of aircraft weight constraints and concerns regarding avionics safety, the use of external monitoring instruments during flight is limited. Therefore, identifying an appropriate ground-based model to simulate +Gz has become a common approach in aviation medicine research. Among the currently available +Gz simulation models, the centrifuge model and the lower body negative pressure (LBNP) model are the most widely used (Schneider et al., 2017; Usselman et al., 2016). The centrifuge model can generate a realistic acceleration environment that closely resembles in-flight conditions and is suitable for both basic research (animal centrifuges) and pilot simulation training (high-G human centrifuges) (Iwasaki et al., 2012; Nordine et al., 2015; Vogt et al., 2014). The LBNP model simulates the physiological effects of fluid redistribution from the head to the lower extremities and is widely used in physiological and psychological research (Goswami et al., 2008; Hinojosa-Laborde et al., 2016).

Due to the high cost and limited availability of high-G human centrifuges, which are primarily used for pilot training, many aviation medicine researchers have turned their attention to the LBNP model. Under the LBNP, blood shifts from the upper body pools in the lower extremities, resulting in a decrease in effective circulating blood volume and cardiac output and an increase in heart rate. This hemodynamic response is similar to the blood redistribution that occurs during +Gz exposure (Baisch et al., 2000; Cooke et al., 2009; Taneja et al., 2007). According to this principle, LBNP can simulate, to some extent, the physiological effects of +Gz on the cardiovascular and cerebrovascular systems, while allowing unrestricted upper-limb movement during experimental tasks. Therefore, the LBNP model is well-suited for investigating changes in human brain function and behavior under simulated +Gz conditions. Research has shown that an LBNP level of −40 mmHg produces hemodynamic effects comparable to those caused by +2 Gz, whereas −50 mmHg simulates cardiovascular responses similar to those observed under +3 ~ 4Gz (Turski et al., 1995). In recent years, with the popularization of noninvasive neuroimaging techniques for cognitive neuroscience research, researchers have gradually begun to focus on the effects of +Gz on individual behavior and brain function.

Because of the serious detrimental effects of G-induced loss of consciousness (G-LOC) resulting from cerebral ischemia, most researchers have focused on the relationship between changes in brain function and autonomic or cardiovascular responses, attempting to reveal the mechanisms underlying the performance impairment during +Gz exposure. Wong et al. (2007) were the first to combine LBNP with functional magnetic resonance imaging (fMRI) to investigate the functional activation of forebrain regions associated with pressure reflexes regulating control of the cardiovascular system during simulated postural hypotension. Subsequently, the researchers compared the functional brain activation under three LBNP levels (−5 mm Hg, −15 mmHg, and −35 mmHg) (Wong et al., 2007). Goswami et al. (2012) examined changes in individual brain function using fMRI during −30 mmHg LBNP (Goswami et al., 2012). Krämer et al. (2014) used PET to evaluate cerebral glucose metabolism in healthy young men exposed to −40 mmHg LBNP. Collectively, these studies demonstrated altered activity in the frontal cortex, thalamus, and cingulate gyrus during LBNP exposure, partially revealing the central nervous mechanism of sympathetic nervous activity abnormalities in the human body under +Gz conditions.

In addition to investigating the association mechanism between brain function and physiological state abnormalities under +Gz stress from a physiological point of view to explain the impaired performance of pilots, researchers have increasingly focused on the association between changes in brain function and cognitive abnormalities under acceleration stress to better understand the effects of +Gz stress on pilot performance. Attention is a core cognitive function that enables pilots to accurately process external stimuli and plays a key role in operational decision-making. Investigating the association between attention-related behavioral changes and alterations in brain function is therefore important for elucidating the neural mechanisms underlying performance changes. Some researchers have done pioneering work in combining neurophysiological techniques and behavioral paradigms to investigate the brain mechanisms of performance changes. Han et al. (2006) recruited 15 healthy young men to complete a visual tracking task under seated LBNP conditions. Middle cerebral artery blood flow velocity and the amplitude and latency of the P300 component were measured before, during, and after exposure to LBNP. The researchers did not find that individual visual tracking errors increased under LBNP conditions, but EEG indices changed significantly with those under normal conditions (Han et al., 2006). Similarly, Cheng et al. (2000) also reported that a prolongation of the P300 latency in individuals under LBNP. These findings suggest that EEG provides high temporal resolution and is more sensitive than behavioral measures for revealing cognitive changes under simulated +Gz conditions.

Previous studies have focused primarily on the later stages of attentional processing—specifically, the response selection stage—while paying relatively little attention to the early stages of perceptual processing. This study aimed to combine behavioral and electroencephalogram (EEG) measures to compare differences in individuals’ attentional capacity between the normal state and the simulated +GZ stress state, thereby characterizing changes in sustained attention during both the early perceptual and late response-selection stages of information processing. The study further aimed to identify brain functional biomarkers capable of distinguishing between the normal state and the simulated +GZ states and provide objective assessment methods and potential biomarkers for pilot selection, physiological monitoring, and other applications.

## 2 Participants and methods

### 2.1 Participants

A total of 16 young male participants were enrolled, with eight assigned to the experimental group and eight to the control group. Participants were19–22 years old, and were right-handed. Eligibility criteria included no history of traumatic brain injury or neurological disease, regular daily routine for the week before the experiment, and no use of drugs or drinks such as coffee. The study was approved by the Ethics Committee of the Air Force Medical Center, and all participants provided their written informed consent before participation.

### 2.2 Methods

#### 2.2.1 Experimental procedure

Simulating positive acceleration stress using an LBNP system, which is mainly used to simulate the phenomenon of fluid transfer to the lower extremities, thereby simulating positive acceleration stress, ultimately leading to cerebral ischemia. Participants in the experimental group completed two experimental sessions involving a sustained attention task. The first session was the baseline assessment, and the participants performed the experiment in a supine position. At least 1 week apart, participants underwent a second assessment under LBNP conditions, with participants lying flat and placed in the LBNP chamber below their waist. The pressure was gradually reduced to −50 mmHg and maintained throughout the completion of experimental tasks. The control group completed two sets of supine position data collection (T1 and T2 rounds), with an interval of at least 1 week between the two collections, similar to the experimental group.

#### 2.2.2 Experimental equipment

The EEG acquisition device is an actiCHamp amplifier and a 32-channel active electrode cap (Brain Products, Brain Products, German) with a sampling rate of 500 Hz. The Cz electrode served as the online reference during data acquisition.

#### 2.2.3 Experimental task

**Figure 1:**
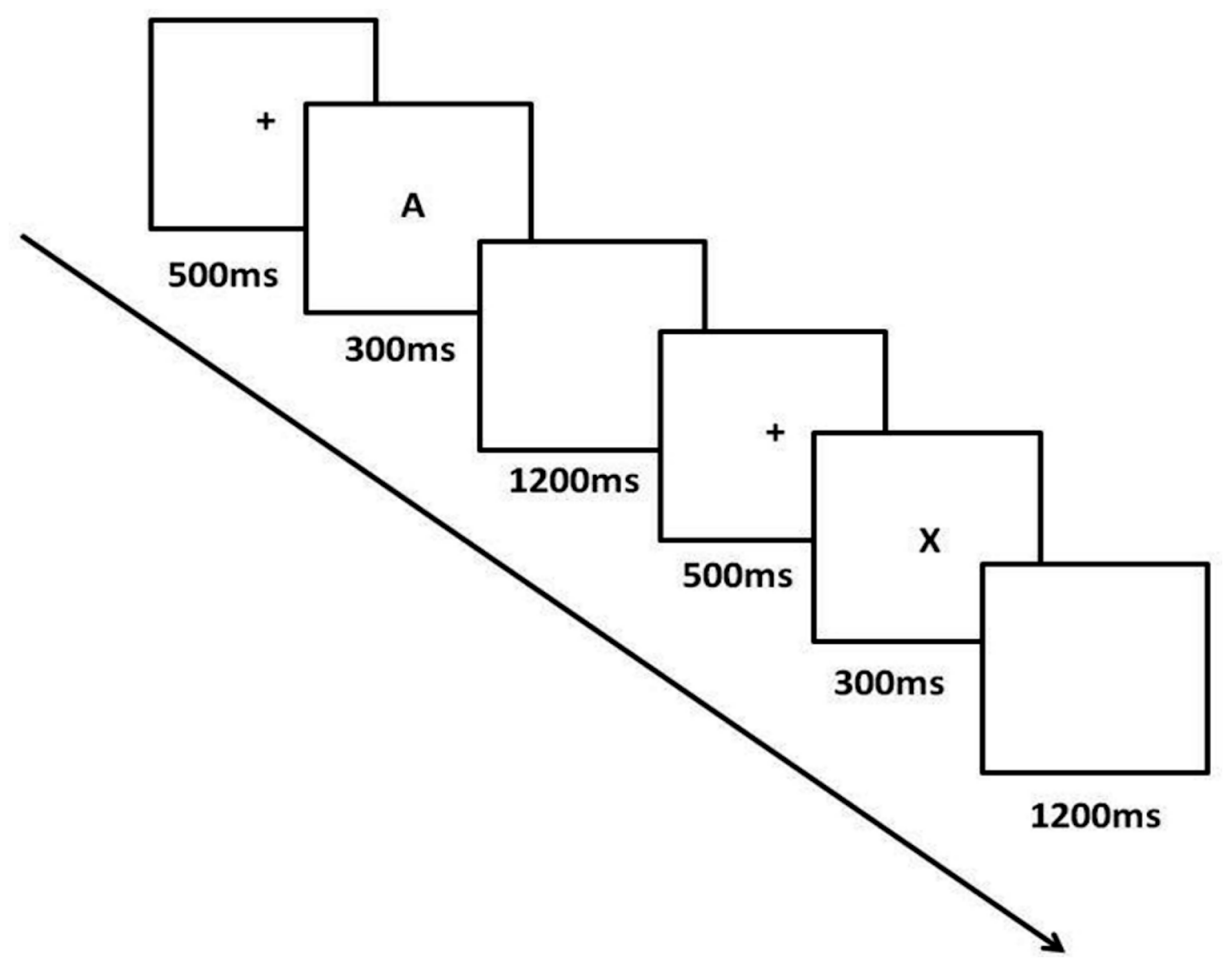
Schematic diagram of continuous attention experiment process.

The assessment of sustained attention is conducted using a continuous performance task (CPT). Each trial began with a black cross fixation point presented at the center of the screen (lasting 500 ms), followed by the presentation of a letter stimulus for 300 ms. Four letter stimuli (A, D, X, and W) were used, each presented 40 times. A blank screen was then displayed for 1,200 ms before the next trial. Among them, the letter X served as the target stimulus (marked as S11), whereas A, D, and W served as non-target stimuli (marked as S12). Participants were instructed to press the left mouse button as soon as possible whenever the target stimuli image “X” appeared and do not need to press a button to respond at other times. Each trial ended with presentation of the fixation cross, after which the subsequent trial commenced (see Figure 1).

### 2.3 EEG data analysis

#### 2.3.1 Preprocessing

(1) Remove useless electrodes: Remove the data of TP9 and TP10 electrodes;
(2) Reference conversion: Convert the Cz reference of the BP32 conductive electrode cap to the brain-wide average reference;
(3) Filtering: filtering with high-pass 1 Hz and low-pass 40 Hz parameters;
(4) Manually remove bad segments and interpolate bad derivatives;
(5) Independent component analysis (ICA) and noise removal: Principal component analysis (PCA) is used for ICA analysis, with a default extraction of 30 components. The EEG and waveform maps of each component are previewed to determine if it is a noise component, and the noise component is manually removed;
(6) Segmentation: Use the stimulus type codes S11 and S12 of each task as segmentation zeros, and take −0.5 to 1.0 s as the segmentation time range;
(7) Baseline correction: Perform baseline correction using prestimuli data as the baseline.

#### 2.3.2 ERP analysis

(1) Individual EEG data averaging: Overlay segmented EEG data of two types of stimuli for each participant in each task to obtain individual event-related potential (ERP) waveforms;
(2) ERP group average: Overlay and average ERP data of all participants in each round to obtain the group average ERP waveform.

#### 2.3.3 Time-frequency analysis

Time-frequency analysis was performed using the short-time Fourier transform and Hamming window, using MATLAB R2018a and custom scripts. After observing the power spectrum images of each electrode over time, the selected frequency band of interest is 8–13 Hz. Using the event occurrence time as the zero point, the selected window of interest is based on the difference time-frequency plots of each experimental task, and the mean power spectrum of the interested frequency band within each time window is calculated for each participant under each condition as the evaluation index.

#### 2.3.4 EEG functional connectivity network construction

**Figure 2:**
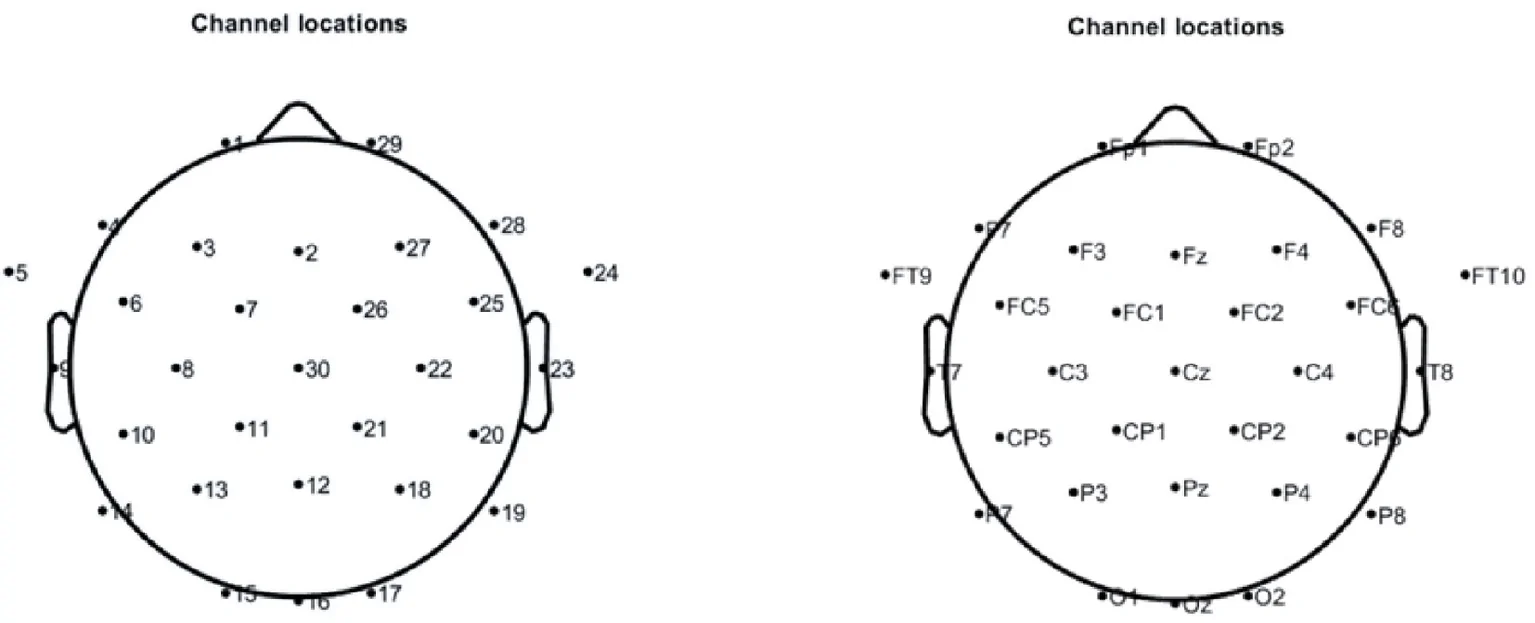
Schematic diagram of EEG electrode numbers and positions.

Using 30 scalp EEG electrodes as nodes, the phase lag index (PLI) and phase-locking value (PLV) were calculated based on EEG time-frequency data as measures of functional connectivity, and these measures were used to construct an EEG functional connectivity network (Figure 2). Functional connectivity networks obtained under the two experimental conditions were compared, and difference networks were generated to visualize condition-related changes.

The phase lag index is a method for detecting the asymmetry of phase difference distribution between two signals and reflects the consistency with which one signal phase leads or lags another signal phase, thereby providing an effective estimate of phase synchronization. The biggest advantage of PLI is that it is insensitive to the volume conductor effect of signals and can only focus on the coupling relationship between signals. The value range of PLI ranges from 0 to 1, and the larger the value, the stronger the phase synchronization between the two signals.

The PLV is another phase-based functional connection method, which actually measures the phase difference between two channel signals. The value range of PLV is from 0 to 1, and the larger the value, the stronger the phase synchronization between the two signals.

### 2.4 Statistical analysis

Statistical analyses were performed using IBM SPSS 20.0, and the Mann–Whitney *U*-test with independent samples (two-tailed) was used to test the difference in behavioral performance changes between the experimental group and the control group in two data collection rounds. The two-tailed Wilcoxon signed-rank test was used to test the difference in ERP/EEG data between the experimental group’s LBNP and baseline rounds. All of the statistical analyses were with a significance level of *p* < 0.05. The *r* value was used as the measure of effect size and was calculated as *r* = *Z*/sqrt(N).

## 3 Results

### 3.1 Behavioral data changes during the sustained attention tasks

The performance data of the sustained attention task behavior of two groups of subjects in different experimental rounds are shown in Table 1. It is worth noting that the standard deviation of various behavioral data of the experimental group participants in the LBNP round increased relative to the baseline round, indicating that there are significant individual differences in the participants’ response to lower body negative pressure stress, and some participants may experience significant fluctuations in behavioral performance data after LBNP stress. In contrast, the standard deviation of the control group’s data in the T2 round was even slightly smaller than that in the T1 round.

**Table 1:** Summary of behavioral data for continuous attention tasks (mean ± standard deviation).

Behavioral data	Experimental group-Baseline	Experimental group-LBNP	Control group-T1	Control group-T1
Accuracy of target stimuli (%)	98.13 ± 2.75	97.25 ± 4.23	98.66 ± 2.66	100.00 ± 0.00
Accuracy of non-target stimuli (%)	98.88 ± 2.48	97.38 ± 4.93	99.11 ± 1.23	99.41 ± 1.10
d’	0.97 ± 0.04	0.95 ± 0.08	0.98 ± 0.03	0.99 ± 0.01
Response time of target stimuli (ms)	359.87 ± 53.93	374.72 ± 62.35	395.31 ± 40.11	397.10 ± 39.91

To compare the differences in behavioral performance changes between the experimental group and the control group in different rounds, the data from the second round of each experiment were subtracted from the data from the first round as the inter-round behavioral performance change indicator, as shown in Table 2.

**Table 2:** Changes in continuous attention task behavior performance of participants between different rounds [median (interquartile range)].

Behavioral data	Experimental group				Control group				*Z*	*p*	*r*
	25th	Median	75th	IQR	25th	Median	75th	IQR			
Change in accuracy of target stimuli (%)	−5.36	0.00	2.68	8.14	0.00	0.00	0.00	2.68	−0.849	0.396	−0.212
Change in accuracy of non-target stimuli (%)	−2.38	0.00	0.00	2.38	0.00	0.00	1.79	1.79	−1.637	0.102	−0.409
Change in d’	−0.06	−0.01	0.03	0.09	0.00	0.01	0.03	0.03	−1.625	0.130	−0.404
Change in response time of target stimuli (ms)	−14.35	17.33	40.30	54.65	−36.65	14.27	30.32	66.98	−0.840	0.401	−0.210

The independent samples nonparametric analysis based on the median values showed no significant differences in changes in various behavioral performance between the experimental and the control groups during the two data collection rounds. However, examination of the 25th percentile values revealed that the response accuracy of the control group during the T2 session was generally equal to or higher than that in T1 round, whereas some participants in the experimental group exhibited a decreasing trend in response accuracy compared to the baseline round in LBNP round (the difference in response accuracy between rounds was negative), indicating that some participants in the experimental group showed trend of impaired sustained attention task processing ability under LBNP stress. From the perspective of data dispersion, the experimental group showed relatively greater fluctuation and variation after LBNP stress, indicating individual differences in the subjects’ tolerance to LBNP stress.

### 3.2 ERP component changes

**Figure 3:**
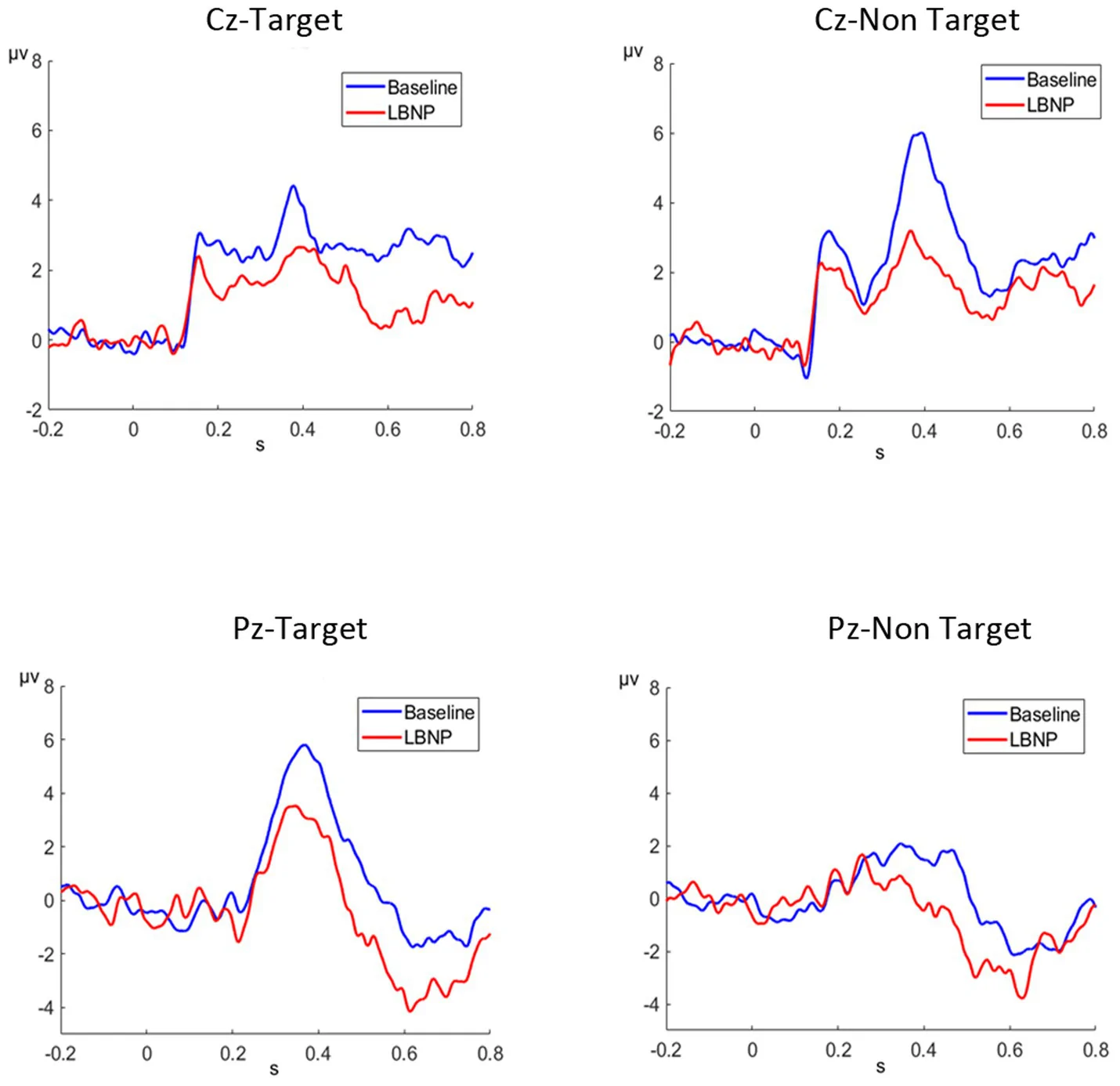
ERPs for Cz and Pz positions across rounds in the sustained attention task.

The superimposed mean plots of ERP components evoked by the target and non-target stimuli in subjects across data collection rounds are shown in Figure 3.

Time windows of 300 ~ 500 ms were selected as the observation window for P300 and 100 ~ 250 ms as the observation window for P2.

The paired-samples Wilcoxon signed-rank test was used to compare the latency data of ERP components in the experimental group between different collection rounds. The results showed that only the latency of the P2 component induced by non-target stimuli at the Cz position significantly differed between the two sessions (Z = −2.243, *p* < 0.05), indicating that the latency of P2 under the LBNP round was earlier than the baseline round. The P2 component is considered an early index of visual attention processing and reflects the ability to classify stimuli. The early P2 latency of non-target stimuli during the LBNP round may reflect improved participants’ early perception and processing ability of such stimuli during the LBNP round. However, behavioral data indicated that the accuracy of non-target stimuli under the LBNP round actually showed a downward trend, which may suggest impaired ability in the later response selection stage of sustained attention tasks (see Table 3).

**Table 3:** Latency of typical ERP components in sustained attention tasks between different rounds [median (interquartile range), ms].

Component -position	Stimuli type	Baseline	LBNP	*Z*	*p*	*r*
P2-Cz	Target stimuli	187.00 (75.00)	171.00 (78.50)	−0.561^b^	0.574	−0.140
	Non-target stimuli	197.00 (55.50)	169.00 (56.00)	−2.243^a^	0.025	−0.561
P300-Cz	Target stimuli	382.00 (78.00)	454.00 (126.50)	−1.120^b^	0.263	−0.280
	Non-target stimuli	387.00 (39.00)	366.00 (49.00)	−1.120^a^	0.263	−0.280
P300-Pz	Target stimuli	375.00 (68.00)	356.00 (98.00)	−0.140^b^	0.889	−0.035
	Non-target stimuli	398.00 (139.50)	391.00 (122.50)	0.000	1.000	0.000

a Represents based on positive rank.

b Represents based on negative rank.

The changes in P300 amplitude at the Cz and Pz positions further confirm this inference, as both target and non-target stimuli showed a trend toward reduced P300 amplitude during the LBNP session. Specifically, the amplitude of P300 induced by non-target stimulation at the Cz position of the lower body negative pressure round was significantly lower than the baseline round amplitude (*Z* = −2.521, *p* < 0.05), and the amplitude of P300 induced by non-target stimulation at the Pz position of the lower body negative pressure round was also significantly lower than the baseline round amplitude (*Z* = −2.380, *p* < 0.05) (see Table 4).

**Table 4:** Amplitudes of typical ERP component for sustained attention tasks between different rounds [median (interquartile range), μv].

Component-position	Stimuli type	Baseline	LBNP	*Z*	*p*	*r*
P2-Cz	Target stimuli	3.63 (4.48)	3.31 (0.95)	−0.840^a^	0.401	−0.210
	Non-target stimuli	4.92 (4.65)	4.28 (1.81)	−1.400^a^	0.161	−0.350
P300-Cz	Target stimuli	5.44 (2.58)	3.46 (2.33)	−1.820^a^	0.069	−0.455
	Non-target stimuli	5.97 (2.72)	4.03 (3.91)	−2.521^a^	0.012	−0.630
P300-Pz	Target stimuli	6.63 (5.38)	4.49 (4.06)	−1.540^a^	0.123	−0.385
	Non-target stimuli	2.84 (3.45)	1.94 (3.18)	−2.380^a^	0.017	−0.595

^a^Represents based on positive rank.

### 3.3 Changes in frequency domain

**Figure 4:**
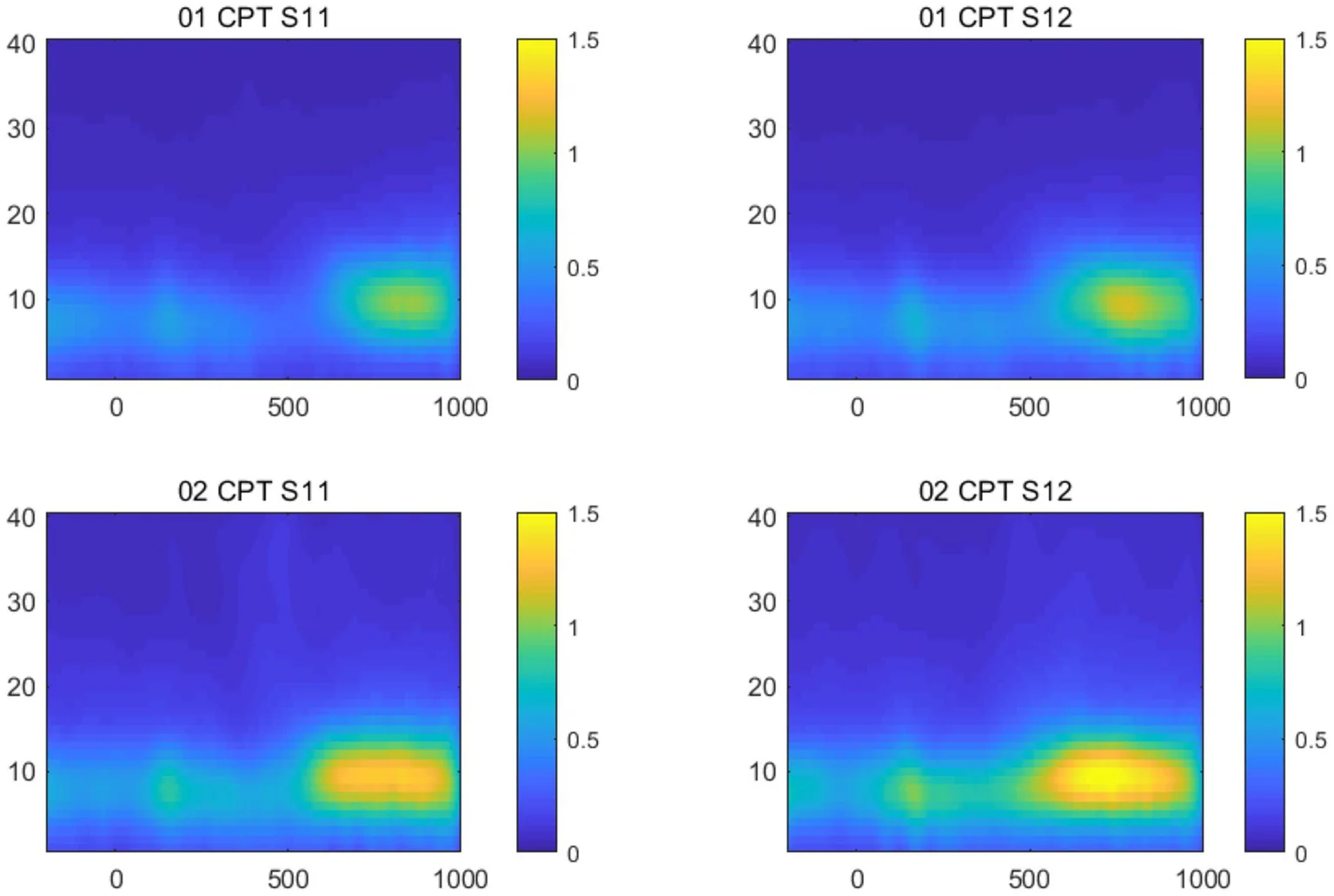
Overall time-frequency average of CPT task (μV^2^/Hz).

The time–frequency maps of EEG induced by two different stimulation conditions (S11-target stimuli and S12-non-target stimuli) in two sessions (baseline round, lower body negative pressure round) are shown in Figure 4. The power spectral values in the figure are the average of 30 electrodes, and the differences are mainly concentrated in the alpha frequency band. In the LBNP round, the power spectrum under S11 and S12 conditions is almost higher than that in the baseline state.

**Figure 5:**
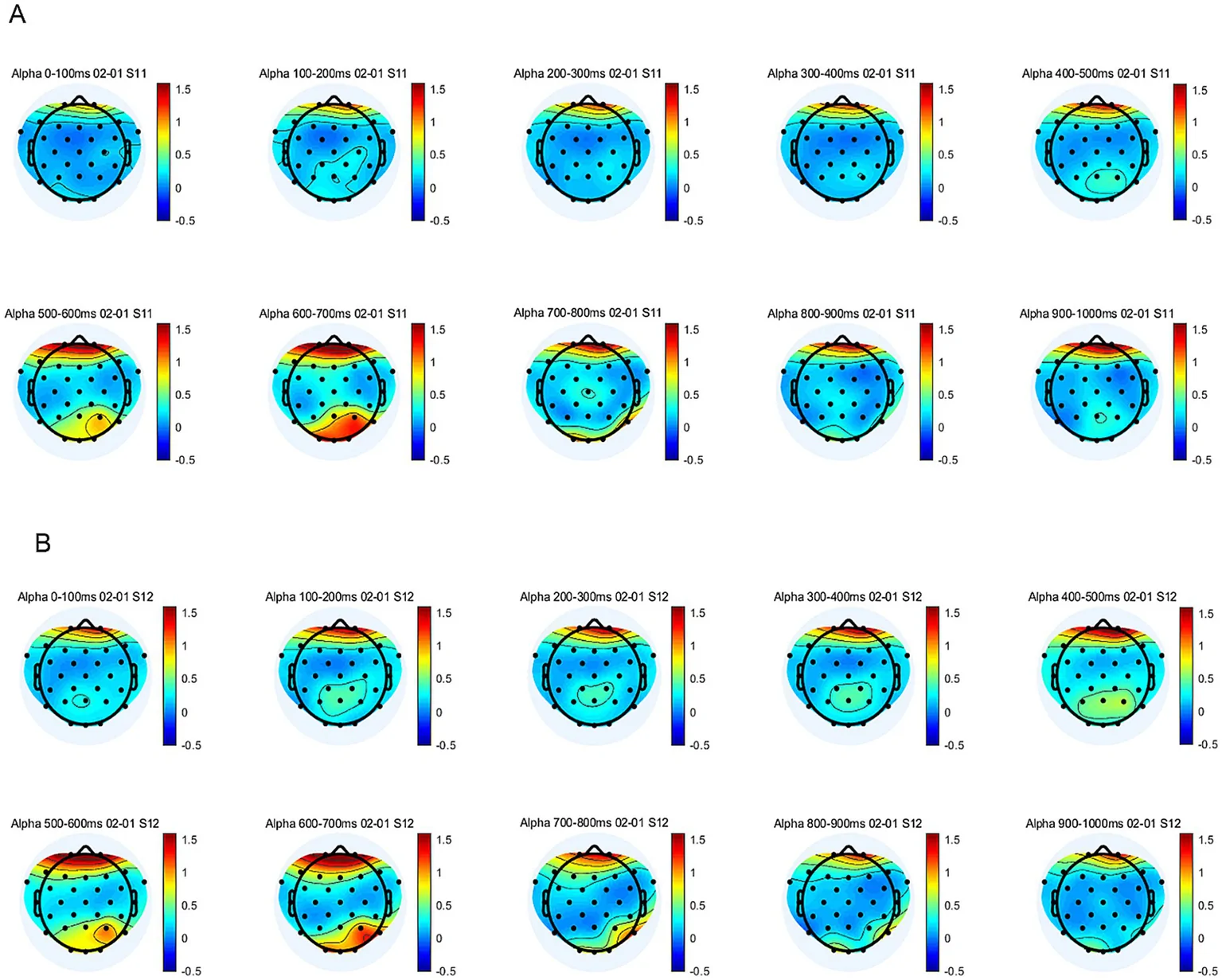
Difference in the mean value of the power spectrum per 100 ms between target and non-target stimuli conditions (μV^2^/Hz). **(A)** Target: LBNP—baseline. **(B)** Non target: LBNP—baseline.

The Wilcoxon signed-ranked test was performed on the mean power spectrum of the alpha frequency band within the time windows of 0 ~ 100 ms, 100 ~ 200 ms, 200 ~ 300 ms, 300 ~ 400 ms, 400 ~ 500 ms, 500 ~ 600 ms, 600 ~ 700 ms, 700 ~ 800 ms, 800 ~ 900 ms, and 900 ~ 1,000 ms after the event occurred. The electrodes with significant differences (baseline round vs. LBNP round) were obtained through paired samples, as shown in Table 5 and Figure 5.

**Table 5:** Electrodes with significant differences in power spectra between two rounds of CPT task Alpha frequency band (*p* < 0.05).

Time window (ms)	Electrode	*Z*	*p*	*p_fdr*	*r*
0-100 ms	Target stimuli (S11)				
	F8	−2.10	0.04	0.26	0.53
	O1	−1.96	0.05	0.26	0.49
	T7	−2.52	0.01	0.18	0.63
	T8	−2.52	0.01	0.18	0.63
	Non-target stimuli (S12)				
	F7	−1.96	0.05	0.22	0.49
	F8	−1.96	0.05	0.22	0.49
	Fp1	−2.24	0.03	0.22	0.56
	Fp2	−1.96	0.05	0.22	0.49
	T7	−1.96	0.05	0.22	0.49
	T8	−1.96	0.05	0.22	0.49
100-200 ms	Target stimuli (S11)				
	F7	−2.10	0.04	0.18	0.53
	F8	−2.10	0.04	0.18	0.53
	Fp1	−2.24	0.03	0.18	0.56
	Fp2	−1.96	0.05	0.19	0.49
	P4	−2.38	0.02	0.18	0.60
	T7	−2.24	0.03	0.18	0.56
	T8	−2.38	0.02	0.18	0.60
	Non-target stimuli (S12)				
	CP6	−2.52	0.01	0.09	0.63
	F3	−2.38	0.02	0.09	0.60
	F4	−2.38	0.02	0.09	0.60
	F7	−2.24	0.03	0.09	0.56
	F8	−2.24	0.03	0.09	0.56
	Fp1	−2.24	0.03	0.09	0.56
	Fp2	−2.24	0.03	0.09	0.56
	Fz	−1.96	0.05	0.12	0.49
	O1	−2.10	0.04	0.10	0.53
	P4	−2.10	0.04	0.10	0.53
	Pz	−2.10	0.04	0.10	0.53
	T7	−1.96	0.05	0.12	0.49
	T8	−2.38	0.02	0.09	0.60
200–300	Target stimuli (S11)				
	CP6	−2.10	0.04	0.36	0.53
	T7	−2.24	0.03	0.36	0.56
	T8	−2.52	0.01	0.35	0.63
	Non-target stimuli (S12)				
	CP6	−2.52	0.01	0.09	0.63
	F3	−2.52	0.01	0.09	0.63
	F7	−1.96	0.05	0.12	0.49
	Fp1	−2.10	0.04	0.11	0.53
	O1	−2.10	0.04	0.11	0.53
	O2	−2.10	0.04	0.11	0.53
	Oz	−2.52	0.01	0.09	0.63
	P3	−2.10	0.04	0.11	0.53
	P4	−2.24	0.03	0.11	0.56
	P8	−2.24	0.03	0.11	0.56
	T7	−1.96	0.05	0.12	0.49
	T8	−2.52	0.01	0.09	0.63
300–400	Target stimuli (S11)				
	F7	−1.96	0.05	0.19	0.49
	F8	−2.10	0.04	0.19	0.53
	Fp1	−2.10	0.04	0.19	0.53
	Fp2	−2.10	0.04	0.19	0.53
	O2	−1.96	0.05	0.19	0.49
	P3	−1.96	0.05	0.19	0.49
	P4	−1.96	0.05	0.19	0.49
	T8	−2.38	0.02	0.19	0.60
	Non-target stimuli (S12)				
	CP6	−1.96	0.05	0.25	0.49
	Fp1	−1.96	0.05	0.25	0.49
	O1	−2.24	0.03	0.25	0.56
	Oz	−2.10	0.04	0.25	0.53
	P3	−2.52	0.01	0.25	0.63
	T8	−2.10	0.04	0.25	0.53
400–500	Target stimuli (S11)				
	FT10	−2.38	0.02	0.17	0.60
	Fp1	−2.10	0.04	0.17	0.53
	Pz	−1.96	0.05	0.17	0.49
	T8	−2.38	0.02	0.17	0.60
	Non-target stimuli (S12)				
	CP5	−1.96	0.05	0.12	0.49
	F8	−1.96	0.05	0.12	0.49
	FC6	−1.96	0.05	0.12	0.49
	Fp1	−2.10	0.04	0.12	0.53
	Fp2	−2.24	0.03	0.11	0.56
	O1	−2.52	0.01	0.11	0.63
	O2	−2.38	0.02	0.11	0.60
	Oz	−2.52	0.01	0.11	0.63
	P4	−2.24	0.03	0.11	0.56
	P7	−1.96	0.05	0.12	0.49
	P8	−2.52	0.01	0.11	0.63
	T8	−2.24	0.03	0.11	0.56
500–600	Target stimuli (S11)				
	CP6	−2.24	0.03	0.08	0.56
	F3	−2.10	0.04	0.08	0.53
	F7	−2.24	0.03	0.08	0.56
	F8	−1.96	0.05	0.09	0.49
	FC1	−2.10	0.04	0.08	0.53
	Fp1	−2.52	0.01	0.08	0.63
	Fp2	−2.10	0.04	0.08	0.53
	Fz	−2.10	0.04	0.08	0.53
	O1	−2.10	0.04	0.08	0.53
	O2	−2.52	0.01	0.08	0.63
	Oz	−2.38	0.02	0.08	0.60
	P3	−1.96	0.05	0.09	0.49
	P4	−2.38	0.02	0.08	0.60
	P8	−2.52	0.01	0.08	0.63
	Pz	−2.10	0.04	0.08	0.53
	T8	−2.10	0.04	0.08	0.53
	Non-target stimuli (S12)				
	CP6	−2.10	0.04	0.10	0.53
	F3	−2.38	0.02	0.10	0.60
	F4	−1.96	0.05	0.12	0.49
	F7	−2.10	0.04	0.10	0.53
	Fp1	−2.10	0.04	0.10	0.53
	Fp2	−2.10	0.04	0.10	0.53
	O1	−2.10	0.04	0.10	0.53
	O2	−2.52	0.01	0.10	0.63
	Oz	−2.52	0.01	0.10	0.63
	P4	−2.52	0.01	0.10	0.63
	P8	−2.10	0.04	0.10	0.53
	T8	−2.10	0.04	0.10	0.53
600–700	Target stimuli (S11)				
	CP6	−2.10	0.04	0.08	0.53
	F3	−2.38	0.02	0.08	0.60
	F4	−1.96	0.05	0.09	0.49
	F7	−2.10	0.04	0.08	0.53
	F8	−1.96	0.05	0.09	0.49
	FC1	−2.52	0.01	0.08	0.63
	FC2	−1.96	0.05	0.09	0.49
	FT10	−2.10	0.04	0.08	0.53
	Fp1	−2.38	0.02	0.08	0.60
	Fz	−2.52	0.01	0.08	0.63
	O1	−2.38	0.02	0.08	0.60
	Oz	−2.24	0.03	0.08	0.56
	P3	−2.10	0.04	0.08	0.53
	P4	−2.10	0.04	0.08	0.53
	P8	−2.24	0.03	0.08	0.56
	Pz	−2.10	0.04	0.08	0.53
	T8	−1.96	0.05	0.09	0.49
	Non-target stimuli (S12)				
	CP6	−1.96	0.05	0.25	0.49
	F3	−2.24	0.03	0.19	0.56
	F7	−2.24	0.03	0.19	0.56
	P4	−2.24	0.03	0.19	0.56
	P8	−2.38	0.02	0.19	0.60
	T8	−1.96	0.05	0.25	0.49
700–800	Target stimuli (S11)				
	CP2	−2.24	0.03	0.13	0.56
	F3	−2.38	0.02	0.13	0.60
	F7	−2.24	0.03	0.13	0.56
	F8	−2.10	0.04	0.13	0.53
	FC1	−2.24	0.03	0.13	0.56
	Fp1	−2.10	0.04	0.13	0.53
	Fp2	−1.96	0.05	0.15	0.49
	Fz	−2.38	0.02	0.13	0.60
	P8	−2.52	0.01	0.13	0.63
	T8	−1.96	0.05	0.15	0.49
	Non-target stimuli (S12)				
	CP6	−1.96	0.05	0.29	0.49
	F7	−1.96	0.05	0.29	0.49
	P4	−2.38	0.02	0.25	0.60
	P8	−2.38	0.02	0.25	0.60
	T8	−2.24	0.03	0.25	0.56
800–900	Target stimuli (S11)				
	F3	−2.38	0.02	0.21	0.60
	F7	−2.10	0.04	0.21	0.53
	FC5	−2.10	0.04	0.21	0.53
	Fp1	−2.10	0.04	0.21	0.53
	P8	−1.96	0.05	0.25	0.49
	T8	−2.10	0.04	0.21	0.53
	Non-target stimuli (S12)				
	F7	−1.96	0.05	0.67	0.49
	T8	−1.96	0.05	0.67	0.49
900–1,000	Target stimuli (S11)				
	C3	−2.10	0.04	0.17	0.53
	Cz	−1.96	0.05	0.17	0.49
	F3	−2.10	0.04	0.17	0.53
	F7	−1.96	0.05	0.17	0.49
	FT10	−2.10	0.04	0.17	0.53
	Fp1	−1.96	0.05	0.17	0.49
	P4	−1.96	0.05	0.17	0.49
	Pz	−2.10	0.04	0.17	0.53
	T8	−2.38	0.02	0.17	0.60
	Non-target stimuli (S12)				
	T8	−2.10	0.04	0.69	0.53

The difference in mean power spectrum between the two rounds is shown below.

To control the risk of false positives due to multiple comparisons, we applied the Benjamini–Hochberg false discovery rate (FDR) correction (*α* = 0.05). After correction, none of the electrode-wise *p*-values reached significance, indicating that, with the current sample size, the differences between the two conditions did not survive the strict statistical threshold. However, when referring to the uncorrected *p*-values (*p* < 0.05), certain combinations of time windows and electrodes showed a consistent pattern of differences in the alpha frequency band, which may reflect either weak effects or insufficient statistical power. Therefore, these findings should be interpreted as suggestive only and not as conclusive evidence.

### 3.4 Changes in functional connections

Nonparametric tests using paired sample data were used to compare the alpha frequency functional connectivity indicators (PLI, PLV) between 0 ~ 1,000 ms of CPT task completion in baseline and LBNP rounds, and no significant differences were found in functional connectivity indicators between the two rounds.

**Figure 6:**
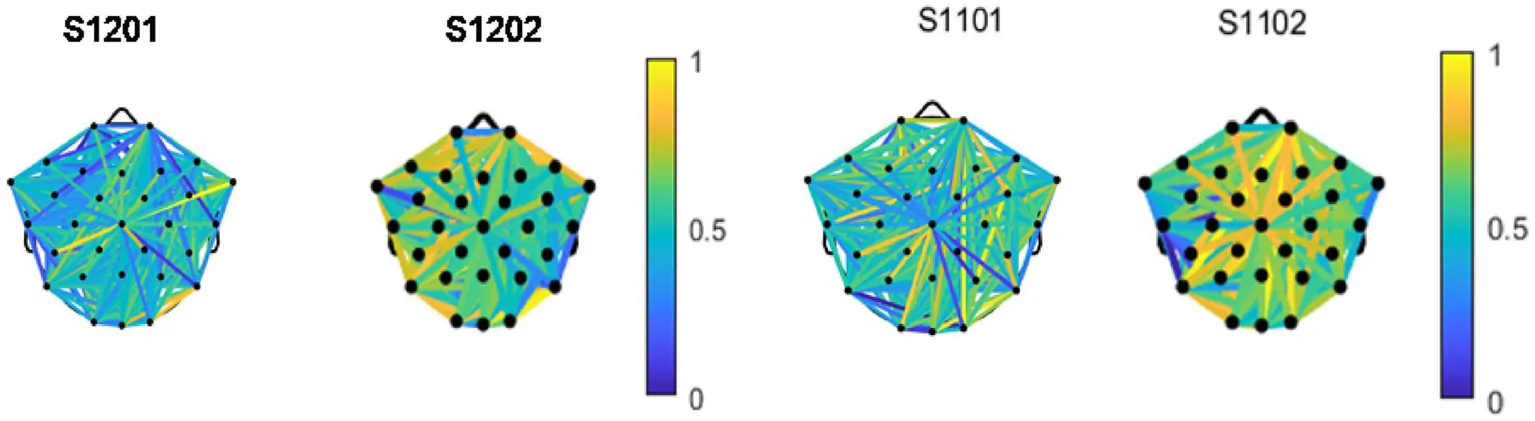
Connection of PLI function in the alpha frequency band of the CPT task.

However, from the functional connectivity diagram of PLI indicators, it can still be seen that the activity synchronization between the electrodes in the alpha frequency band under LBNP shows an increasing trend compared to baseline, as shown in Figure 6. This is consistent with the trend of power spectrum values on the frequency domain indicators described earlier.

## 4 Discussion

In the current study, behavioral data did not reveal a statistically significant impairment in sustained attention following LBNP treatment; however, the findings suggested a trend toward impaired late-stage response selection. It is possible that enhanced early perceptual processing partially compensated for effects of impaired late-stage response selection, resulting in only limited impairment in overall behavioral performance.

EEG measures were more sensitive than behavioral measures. The amplitude and latency data of ERP components reflected significant changes in participants’ sustained attention capacity under LBNP conditions, indicating improved early perceptual processing ability and reduced late-stage response selection. These neural changes were accompanied by a downward trend in behavioral performance on the sustained attention task under LBNP conditions compared to baseline, thereby confirming the hypothesis derived from the behavioral data. These results were similar to the study by Han et al., which may indicate that behavioral data are not sensitive enough to reflect the impaired ability of visual information processing under LBNP (Han et al., 2006).

The dissociation between early- and late-stage attentional processing observed in this study is a highly intriguing finding that fully demonstrates the temporal precision of EEG. EEG can distinguish between different subcognitive processing stages that cannot be differentiated at the behavioral level. The enhancement of early perceptual processing ability under LBNP conditions may represent an adaptive regulatory strategy of the brain that helps maintain survival capacity under stress. When a decline in late response selection ability becomes inevitable, enhancing early perceptual processing ability can partially offset the detrimental effects, thereby maintaining the individual’s overall responsiveness to external stimuli at a certain level and preventing excessive detrimental effects from severely impacting sustained attention performance. In the previous study, Wang et al. (2002) reported reduced P300 amplitude during a visual oddball task under 50 mmHg of LBNP, which is consistent with the present findings. Those results indicated that a decline in ability in the late response selection stage under LBNP is a credible finding. The P2 component is thought to reflect early perceptual processing of visual information (Crowley and Colrain, 2004; Daffner et al., 2015). The change in P2 under LBNP was not noticed in the previous studies; the enhanced early perceptual processing ability reflected by P2 was found in the current study, which expands the understanding of the sub-processing of sustained attention under LBNP.

In addition, EEG time–frequency analysis in the current study also found the damaging effect of LBNP on sustained attention processing ability. Previous studies have shown that the consistency of alpha-band neuron activity generally decreases when individuals engage in cognitive tasks (Foxe and Snyder, 2011; Samaha and Postle, 2015; Van Diepen et al., 2019). In the present study, the activity consistency of alpha-frequency neurons was enhanced under LBNP, indicating a decrease in cognitive resource investment in sustained attention tasks for individuals, consistent with the ability impairment changes reflected in ERP data. From a time-window perspective, this impairment effect is strongest after 500 ms of visual stimulation presentation. It is also in the late processing stage, like the time window of impaired response selection ability found in ERP, which is also in the late processing stage. This evidence confirms that the damaging effect of LBNP on sustained attention ability is mainly concentrated in the late processing stage.

Based on the comprehensive results, there is a clear damaging effect of LBNP on sustained attention ability, which is mainly concentrated in the late processing stage of response selection to stimuli. With the enhancement of early perception processing ability toward stimuli, the damaging effect may be partially offset. Ultimately, from the perspective of behavioral performance, individuals’ sustained attention processing performance can still be maintained at a certain level under +3 ~ 4Gz stress, enabling individuals to perceive external information stimuli relatively timely and accurately under stressful conditions, to maintain a certain level of survival ability.

## 5 Conclusion

In the present study, it was found that there is a damaging effect on individuals’ sustained attention processing ability under simulated +Gz stress. The amplitude or latency of ERP and EEG power spectrum can be used as quantitative indicators to observe the damaging effect. In addition, there is a separation effect between the early and late stages of visual attention processing, reflecting the body’s adaptive adjustment ability in response to stress. On the one hand, the discovery of damage effects and their quantitative indicators can potentially be used in pilot selection tasks to assist in assessing individual acceleration endurance and understanding the sub differences between individuals with the same level of acceleration endurance. Based on the performance impairment and EEG changes of individuals under LBNP, the level of brain function maintenance under cerebral ischemia can be quantitatively understood. Conversely, the separation effect of visual attention processing in the early and late stages discovered in the current study, especially the compensatory enhancement of early perceptual processing ability, can provide some new perspectives for the maintenance or rehabilitation of cognitive function in patients with cerebral ischemic diseases to a certain extent. However, because of the limited sample size in the current study, the findings should be considered exploratory in nature. Nevertheless, they offer valuable insights into changes in attentional capacity under acceleration stress. Future research should examine a broader range of LBNP levels and include larger cohorts to provide more robust evidence.

## Data Availability

The raw data supporting the conclusions of this article will be made available by the authors, without undue reservation.

## References

[ref1] Bacevic N. Ninkovic M. Drvendzija M. Vidakovic J. Bacevic M. Stepanic P. (2024). Heart rate variability as a predictor of +G(z) tolerance during the high-G selective test. Aerosp. Med. Hum. Perform 95, 93–100. doi: 10.3357/AMHP.6319.2024, 38263102

[ref2] Baisch F. Beck L. Blomqvist G. Wolfram G. Drescher J. Rome J. L. . (2000). Cardiovascular response to lower body negative pressure stimulation before, during, and after space flight. Eur. J. Clin. Investig. 30, 1055–1065. doi: 10.1046/j.1365-2362.2000.00750.x, 11122320

[ref3] Biernacki M. P. Tarnowski A. Lengsfeld K. Lewkowicz R. Kowalczuk K. Dereń M. (2013). +Gz load and executive functions. Aviat. Space Environ. Med. 84, 511–515. doi: 10.3357/ASEM.3224.2013, 23713217

[ref4] Cheng Y. Jiao Z. Yan G. Jin Z. (2000). The characteristics of cerebral functional indices during occurrence of presyncopal symptom induced by lower body negative pressure. Chin. J. Aerosp. Med. 11, 145–148.

[ref5] Clément G. Moore S. T. Raphan T. Cohen B. (2001). Perception of tilt (somatogravic illusion) in response to sustained linear acceleration during space flight. Exp. Brain Res. 138, 410–418. doi: 10.1007/s002210100706, 11465738

[ref6] Cooke W. H. Rickards C. A. Ryan K. L. Kuusela T. A. Convertino V. A. (2009). Muscle sympathetic nerve activity during intense lower body negative pressure to presyncope in humans. J. Physiol. 587, 4987–4999. doi: 10.1113/jphysiol.2009.177352, 19703962 PMC2770161

[ref7] Croft R. J. Kölegård R. Tribukait A. Taylor N. A. S. Eiken O. (2021). Effects of acceleration-induced reductions in retinal and cerebral oxygenation on human performance. Aerosp Med. Hum. Perform. 92, 75–82. doi: 10.3357/AMHP.5731.2021, 33468287

[ref8] Crowley K. E. Colrain I. M. (2004). A review of the evidence for P2 being an independent component process: age, sleep and modality. Clin. Neurophysiol. 115, 732–744. doi: 10.1016/j.clinph.2003.11.021, 15003751

[ref9] Daffner K. R. Alperin B. R. Mott K. K. Tusch E. S. Holcomb P. J. (2015). Age-related differences in early novelty processing: using PCA to parse the overlapping anterior P2 and N2 components. Biol. Psychol. 105, 83–94. doi: 10.1016/j.biopsycho.2015.01.002, 25596483 PMC4374636

[ref10] Fong K. L. Fan S. W. (1997). An overview of the physiological effects of sustained high +Gz forces on human being. Ann. Acad. Med. Singap. 26, 94–103. 9140585

[ref11] Foxe J. J. Snyder A. C. (2011). The role of alpha-band brain oscillations as a sensory suppression mechanism during selective attention. Front. Psychol. 2:154. doi: 10.3389/fpsyg.2011.00154, 21779269 PMC3132683

[ref12] Goswami R. Frances M. F. Steinback C. D. Shoemaker J. K. (2012). Forebrain organization representing baroreceptor gating of somatosensory afferents within the cortical autonomic network. J. Neurophysiol. 108, 453–466. doi: 10.1152/jn.00764.2011, 22514285

[ref13] Goswami N. Loeppky J. A. Hinghofer-Szalkay H. (2008). LBNP: past protocols and technical considerations for experimental design. Aviat. Space Environ. Med. 79, 459–471. doi: 10.3357/ASEM.2161.2008, 18500042

[ref14] Han W. Q. Hu W. D. Wen Z. H. Ma R. S. Tian R. F. (2006). Change of cerebral blood flow velocity and event related potential and comparison of different stimulus paradigms under lower body negative pressure. J. Fourth Mil. Med. Univ. 27, 2199–2202.

[ref15] Hinojosa-Laborde C. Howard J. T. Mulligan J. Grudic G. Z. Convertino V. A. (2016). Comparison of compensatory reserve during lower-body negative pressure and hemorrhage in nonhuman primates. Am. J. Physiol. Regul. Integr. Comp. Physiol. 310, R1154–R1159. doi: 10.1152/ajpregu.00304.2015, 27030667 PMC5504419

[ref16] Iwasaki K. Ogawa Y. Aoki K. Yanagida R. (2012). Cerebral circulation during mild +Gz hypergravity by short-arm human centrifuge. J. Appl. Physiol. 112, 266–271. doi: 10.1152/japplphysiol.01232.2011, 22052869

[ref17] Jia H. Cui G. Xie S. Tian D. Bi H. Guo S. (2009). Vestibular function in military pilots before and after 10 s at +9 Gz on a centrifuge. Aviat. Space Environ. Med. 80, 20–23. doi: 10.3357/ASEM.2186.2009, 19180854

[ref18] Kim S. Cho T. Lee Y. Koo H. Choi B. Kim D. (2017). G-LOC warning algorithms based on EMG features of the gastrocnemius muscle. Aerosp. Med. Hum. Perform. 88, 737–742. doi: 10.3357/AMHP.4781.2017, 28720183

[ref19] Krämer H. H. Ament S. J. Breimhorst M. Klega A. Schmieg K. Endres C. . (2014). Central correlation of muscle sympathetic nerve activation during baroreflex unloading—a microneurography-positron emission tomography study. Eur. J. Neurosci. 39, 623–629. doi: 10.1111/ejn.12437, 24528135

[ref20] McKinley R. A. Tripp L. D. Jr. Fullerton K. L. Goodyear C. (2013). Sustained acceleration on perception of relative position and motion. Aviat. Space Environ. Med. 84, 184–189. doi: 10.3357/ASEM.3369.2013, 23513278

[ref21] McKinlly R. A. Gallimore J. J. (2013). Computational model of sustained acceleration effects on human cognitive performance. Aviat. Space Environ. Med. 84, 780–788. doi: 10.3357/ASEM.2584.2013, 23926652

[ref22] Metzler M. M. (2020). G-LOC due to the push-pull effect in a fatal F-16 mishap. Aerosp. Med. Hum. Perform. 91, 51–55. doi: 10.3357/AMHP.5461.2020, 31852575

[ref23] Nordine M. Maggioni M. A. Stahn A. Mendt S. Brauns K. Gunga H.-C. . (2015). Form influences function: anthropometry and orthostatic stability during sustained acceleration in a short arm human centrifuge. Acta Astronaut. 115, 138–146. doi: 10.1016/j.actaastro.2015.05.025

[ref24] Pollock R. D. Britton J. K. Green N. D. C. Hendriksen D. Hodkinson P. D. Anderton R. A. . (2024). Prevention of G-induced effects on vision and consciousness during simulated suborbital spaceflight. Aerosp. Med. Hum. Perform. 95, 897–901. doi: 10.3357/AMHP.6511.2024, 39711414

[ref25] Rickards C. A. Newman D. G. (2005). G-induced visual and cognitive disturbances in a survey of 65 operational fighter pilots. Aviat. Space Environ. Med. 76, 496–500. 15892551

[ref26] Ryoo H. C. Sun H. H. Shender B. S. Hrebien L. (2004). Consciousness monitoring using near-infrared spectroscopy (NIRS) during high +Gz exposures. Med. Eng. Phys. 26, 745–753. doi: 10.1016/j.medengphy.2004.07.003, 15564111

[ref27] Samaha J. Postle B. R. (2015). The speed of alpha-band oscillations predicts the temporal resolution of visual perception. Curr. Biol. 25, 2985–2990. doi: 10.1016/j.cub.2015.10.007, 26526370 PMC4654641

[ref28] Sandor P. M. Pellieux L. Godfroy M. Ossard G. Dancer A. (2004). Hearing thresholds during Gz acceleration with masking noise. Aviat. Space Environ. Med. 75, 952–955. 15558994

[ref29] Schneider S. Zander V. Vogt T. Abeln V. Strüder H. K. Jacubowski A. . (2017). Hemodynamic and neuroendocrinological responses to artificial gravity. Gravit. Space Res. 5, 80–88. doi: 10.2478/gsr-2017-0012

[ref30] Taneja I. Moran C. Medow M. S. Glover J. L. Montgomery L. D. Stewart J. M. (2007). Differential effects of lower body negative pressure and upright tilt on splanchnic blood volume. Am. J. Physiol. Heart Circ. Physiol. 292, H1420–H1426. doi: 10.1152/ajpheart.01096.2006, 17085534 PMC4517828

[ref31] Truszczynski O. Lewkowicz R. Wojtkowiak M. Biernacki M. P. (2014). Reaction time in pilots during intervals of high sustained g. Aviat. Space Environ. Med. 85, 1114–1120. doi: 10.3357/ASEM.4009.2014, 25329944

[ref32] Truszczynski O. Wojtkowiak M. Lewkowicz R. Biernacki M. P. Kowalczuk K. (2013). Reaction time in pilots at sustained acceleration of +4.5 G(z). Aviat. Space Environ. Med. 84, 845–849. doi: 10.3357/ASEM.3490.2013, 23926661

[ref33] Turski B. K. Kuzak W. Debinski W. B. Gembicka-Kuzak D. M. Dabrowski O. B. (1995). Application of multivariate statistical analysis to estimate +Gz tolerance based on the changes of hemodynamic parameters during lower body negative pressure (LBNP). J. Gravit. Physiol. 2, 35–36. 11538924

[ref34] Usselman C. W. Nielson C. A. Luchyshyn T. A. Gimon T. I. Coverdale N. S. Van Uum S. H. . (2016). Hormone phase influences sympathetic responses to high levels of lower body negative pressure in young healthy women. Am. J. Physiol. Regul. Integr. Comp. Physiol. 311, R957–R963. doi: 10.1152/ajpregu.00190.2016, 27733385 PMC5130577

[ref35] Van Diepen R. M. Foxe J. J. Mazaheri A. (2019). The functional role of alpha-band activity in attentional processing: the current zeitgeist and future outlook. Curr. Opin. Psychol. 29, 229–238. doi: 10.1016/j.copsyc.2019.03.015, 31100655

[ref36] Vogt T. Abeln V. Strüder H. K. Schneider S. (2014). Artificial gravity exposure impairs exercise-related neurophysiological benefits. Physiol. Behav. 123, 156–161. doi: 10.1016/j.physbeh.2013.10.020, 24184512

[ref37] Wang L. Han W. Ma R. Cheng H. Heying N. (2002). Effects of lower body negative pressure in upright seated position on human brain function. Med. J. Chin. People's Armed Pol. Forc. 10, 587–590.

[ref38] Wong S. W. Kimmerly D. S. Massé N. Menon R. S. Cechetto D. F. Shoemaker J. K. (2007). Sex differences in forebrain and cardiovagal responses at the onset of isometric handgrip exercise: a retrospective fMRI study. J. Appl. Physiol. 103, 1402–1411. doi: 10.1152/japplphysiol.00171.2007, 17615282

